# Hyperacusis in children: a scoping review

**DOI:** 10.1186/s12887-020-02223-5

**Published:** 2020-06-29

**Authors:** Iskra Potgieter, Kathryn Fackrell, Veronica Kennedy, Rosa Crunkhorn, Derek J. Hoare

**Affiliations:** 1 National Institute for Health Research (NIHR) Nottingham Biomedical Research Centre, University of Nottingham, Ropewalk House, 113 The Ropewalk, Nottingham, NG1 5DU UK; 2 Otology and Hearing Group, Division of Clinical Neuroscience, School of Medicine, University of Nottingham, Nottingham, NG7 2UH UK; 3 National Institute for Health Research, Evaluation, Trials and Studies Coordinating Centre (NETSCC), University of Southampton, Southampton, UK; 4 4 Paediatric Audiology Department, Bolton NHS Foundation Trust, Bolton, UK

**Keywords:** Hyperacusis, Children, Clinical profile, Assessment, Treatment, Scoping review

## Abstract

**Background:**

Hyperacusis is a chronic condition commonly defined as a lowered tolerance or increased sensitivity to everyday environmental sounds. It has been viewed as a paediatric disorder which can cause significant impairment to a child’s normal functioning. Although clinical guidance highlights the importance of identifying whether the child has intolerance to loud sounds and managing this appropriately, there are currently no assessment or treatment methods that have been designed and tested for use with children with hyperacusis. A review is therefore indicated to consider the profile of children with hyperacusis as a basis for future research into their assessment and treatment.

**Method:**

A scoping review methodology was followed with literature searches conducted in Embase, PsychINFO, PubMed CENTRAL, Scopus, Web of Science and Google Scholar. Research articles were included if they reported on research studies describing children diagnosed with hyperacusis, providing clinical profile information, and/or reporting on an assessment or management method for children with a primary complaint of hyperacusis. Data were charted on Excel and verified by a second researcher. Twenty-one research articles were included.

**Results:**

Children with hyperacusis are typically described in terms of age at presentation, troublesome sounds, physical sensation, behavioural reactions, coping strategies, comorbid conditions and impact on daily life. Methods of assessing the children include semi-structured interviews, questionnaires, neurological assessment, observation and uncomfortable loudness levels. Management methods include psychological therapy, sound therapy, tinnitus retraining therapy, medication and neuro-rehabilitation.

**Conclusion:**

The information we catalogued on various elements of clinical profile, assessment and management can serve as a stepping stone in future research developing questionnaires for clinical measurement of the impact of hyperacusis on children, and the measurement of treatment related change in clinic and in trials. Positive outcomes were noted by the authors following all of the above treatments; future research must compare these and specify the parameters for optimal results.

## Background

Hyperacusis is a chronic condition commonly defined as a lowered tolerance or increased sensitivity to everyday environmental sounds [1, 2]. It can cause significant impairment to a person’s normal functioning. Conditions such misophonia and phonophobia also involve decreased sound tolerance and can be co-existing with hyperacusis, evoking similar reactions and potentially involving the same brain areas of emotion and fear [3, 4]. That said, there is no definitive evidence as to the aetiology and diagnosis of hyperacusis as yet, therefore differentiating it from these conditions is debatable. According to the evidence to date, phonophobia may differ from hyperacusis in that it is a psychiatric condition, diagnosable under the DSM-IV classification for specific phobias [5] and which involves fear from the troublesome sound(s). Misophonia has been commonly associated with difficult to control bursts of anger and sometimes rage when exposed to specific human oral and nasal sounds [6, 7]. Hyperacusis is also distinct from recruitment, which involves a narrowing of the auditory dynamic range due to hearing loss [8]. Instead, people with hyperacusis tend to experience intense discomfort or pain due to certain sounds of various loudness levels - from hushed sounds such as distant traffic or the sound of a refrigerator motor, to loud sounds such as hand dryers or electric food mixers. Patients would perceive these sounds as much louder or intense than they actually are [9]. Thus, patients can either have normal uncomfortable loudness levels (ULLs), except for specific troublesome sounds, or have generally reduced ULLs, irrespective of the type of sound they are exposed to [10]. People with hyperacusis also exhibit normal hearing (for their age) or slight hearing loss on pure tone audiograms, and reportedly 86% experience tinnitus [11, 12]. Hyperacusis can occur in otherwise healthy individuals [13] but is generally associated with a number of pathologies, including closed head trauma [14], depression [15], and post-traumatic stress [16]. Hyperacusis is also a complaint in approximately 95% of people with William’s Syndrome (WS), and 63% of people with autism spectrum disorders (ASD) [17, 18]. It has also been linked to Asperger’s Syndrome (AS) [19], though AS falls under the umbrella diagnosis of ASD. The high incidence of hyperacusis in developmental disorders has led to hyperacusis being viewed as a typically paediatric disorder [10, 20], although it also occurs in children with no other health concerns. Evidence on the prevalence of hyperacusis in children is sparse and inconclusive. A recent systematic review by Rosing et al. [21] documented that this can vary between 3.2 and 17.1%, the variability likely a product of how the prevalence questions were posed in different studies. However, hyperacusis is clearly a significant clinical concern in children. The British Society of Audiology (BSA) clinical practice guidance for tinnitus in children [22] highlights the importance of identifying whether the child has any intolerance of loud sounds, and managing this appropriately. However, no clinical guidelines specifically for hyperacusis in children have yet been developed [23]. A review is therefore indicated to consider the profile of children with hyperacusis and the priorities for future research.

### Aims

This scoping review aimed to catalogue reports on children who experience hyperacusis with a focus on identifying a clinical profile of the condition. Hence, the primary research question was to identify the range of symptoms in terms of physical sensations, reactions, coping behaviours, and areas of impairment in daily living encountered by children. The secondary research question was to document the published methods used by clinicians and researchers to diagnose, assess, and treat hyperacusis in children.

## Methods

Due to the broad, explorative nature of our research questions in this emerging field of evidence, a scoping review was determined to be the most suitable methodology. Unlike the systematic review which aims to answer specific questions in a rigorous manner, the scoping review is designed to allow the mapping of key concepts that underpin a research topic [24], which, in this case was the clinical profile of children with hyperacusis. Hence a methodological framework for scoping reviews was followed [24–27] involving six stages whereby (1) the purpose and research questions were defined, (2) relevant studies were identified using a three-step literature search to balance breadth and comprehensiveness, (3) studies were selected using an iterative team approach, (4) data were charted, (5) results were collated, summarised and reported, including the implications for policy, practice, or research, and (6) findings were reviewed by clinical experts in the field who did not take part in stages 1–5. This scoping review does not include a previously published protocol.

### Eligibility criteria

Records were included if they focused on paediatric (under 18 years old) patients with a complaint of hyperacusis, and provided clinical profile information. This included data on hyperacusis-related symptoms or reactions, type of bothersome sounds, coping behaviours, areas/level of dysfunction. Records reporting methods of assessing hyperacusis, and/or treatment of hyperacusis with outcomes in paediatric populations were also included. Records were required to be peer reviewed or grey literature, reporting randomised or non-randomised trials, cohort/ retrospective studies, case studies, case series, or expert clinical opinions (where child cases were described).

Records were excluded if they focused on patients over 18 years of age; reported on tinnitus, recruitment, phonophobia, misophonia or other conditions only (without hyperacusis); were reviews (including systematic reviews), patents, animal studies, or studies not available in English; or were patient reports or information posted on social media or internet forums or blogs. Records containing insufficient amount of data to extract were also excluded.

### Information sources and search strategy

A limited search was first conducted in Embase, Ovid, and PubMed to check that the search terms derived optimal results. Official searches were then conducted in PubMed; Embase; PsycINFO; Scopus; Cumulative Index to Nursing and Allied Health Literature (CINAHL); Web of Science; ProQuest Dissertations and Theses. Google and Google Scholar were searched until a saturation point was reached when one page of consecutive search results contained no entries relevant to the aims of the review.

We applied a specific search term strategy in each search engine using the following search terms: hyperacus* or auditory hypersthesia* or sound intolerance or uncomfortable loud* or reduced sound tolerance or lowered sound tolerance or sound hypersensitivity or sound sensitivity and child* or infant* or pediat* or paediat* or student* or pupil*. We searched in article topics, titles, and/or abstracts. Where possible, filters were applied to retrieve articles in the English language and with human participants only. There was no restriction on the search period. Finally, hand searches were conducted including the reference lists of relevant literature reviews, research articles, and selected journals determined using the interquartile rule for outliers. Initial searches were conducted in February 2018 and updated in May 2020.

### Study selection

Using an iterative approach, all abstracts were assessed independently by two researchers as meeting the review criteria or not. Records were first screened by title and abstract and in the next stage - by full text. When disagreements regarding the inclusion or exclusion of a record arose, the two reviewers discussed their rationale until agreement was reached, or a third reviewer was consulted to adjudicate.

### Charting the data

A bespoke data charting form was developed. This is a spreadsheet on Excel containing the items of data that were extracted from each record. Data items for charting included year and country of publication, clinician providing care (i.e. type of clinician, for example, a psychologist, or clinical setting, for example, ENT), study type (e.g. case study, cohort, survey), sample size, age, onset age, gender, comorbidities (other medically diagnosed disorders), assessments (e.g. questionnaires, semi-structured interview), uncomfortable loudness levels (ULLs), troublesome noises (as reported by the child), physical sensation (i.e. the nature of physical discomfort experienced by the patient when hearing the troublesome sound), reaction (i.e. associated emotional or behavioural reaction by the child when hearing the troublesome sounds), coping behaviours (strategies used by the child or his/her parents to manage hyperacusis), impact on daily life (the limitations caused by hyperacusis on the child’s and their family’s normal life), author’s summary of complaint (e.g. hyperacusis or reduced tolerance to sound), treatment (specifically aimed at hyperacusis), and treatment outcome (relative to hyperacusis only). This charting form was piloted on two records and the process and data items were discussed before commencing the full data extraction procedure. Two researchers (IP and DH) charted the data from each included record. All data were charted verbatim from the records. No critical appraisal of included sources of evidence was conducted. The accuracy of data charting was verified by a second reviewer (DH or KF). The data from Excel was then grouped verbatim on Word according to the research questions.

## Results

**Fig. 1:**
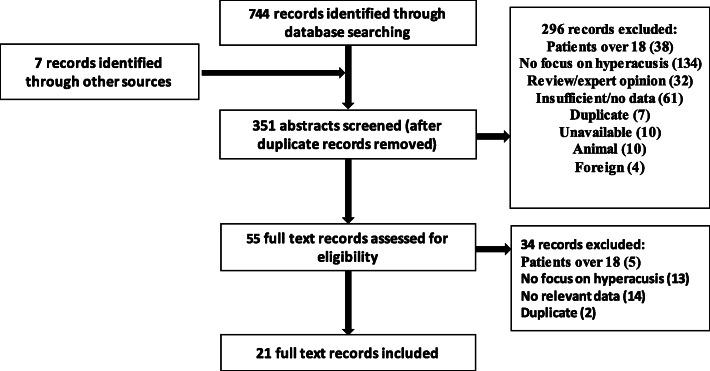
Flow chart of records

Figure 1 displays the flow of records identified, screened, included or excluded, and reasons for exclusion. We identified 744 records through database searching and seven from hand searching. After removing duplicates, we were left with 351 records, which were screened at a title and abstract level. Of these, 55 were selected for full text screening, following which, 34 were excluded and 21 records were deemed eligible for the review (see Table 1).

**Table 1:** Descriptive summary of included records

1st Author, Year, Country	Design	Sample size, age, gender	Care provider	Complaint
[40] Aazh 2011 UK	Case study	1,15 yrs., M	NS	Hyperacusis
[32] Aazh 2018 UK	Retrospective cohort study	62, 4-18yrs., MF	Tinnitus and hyperacusis therapy specialist clinic	Sound intolerance
[36] Adanir 2017 Turkey	Case study	1, 11 yrs., M	Child and adolescent psychiatry	Auditory hypersensitivity
[28] Amir 2018 UK	Retrospective cohort study	412, median 7 yrs., MF	Paediatric ENT/ paediatric hyperacusis service	Hyperacusis
[34] Borawska 2004 Poland	Retrospective case review	11, 5-18yrs., MF	Tinnitus clinic	Hyperacusis
[44] Coelho 2007 USA	Cross-over study	16, 5-12 yrs., MF	NS	Bothered and annoyed by sounds
[41] Esposito 2016 USA	Case study	1, 7 yrs., F	NS	Hyperacusis
[37] Ghandizeh 2009 Iran	Case study	1, 5 yrs., F	Psychiatry	Very sensitive to sounds
[20] Hall 2016 UK	Prospective cohort study	261, 11 yrs., MF	NS	Oversensitive to sound
[43] Janes 2014 UK	Observational study	21, 6-15 yrs., MF	NS	Auditory hypersensitivity
[2] Kennedy 2018 UK	Case study	2, 6 yrs., 9 yrs., M	NS	Case 1: Difficulty tolerating noise at achool; case 2: pain and fear in response to loud sounds
[33] Myne 2018 UK	Retrospective case review	61, NS, MF	Paediatric audiology	Hyperacusis
[29] Nigam 1994 UK	Case study	1, 21mnths, F	ENT clinic	Distress in response to ordinary sounds
[38] O’Reilly 2000 Ireland	Case study	1, 5 yrs., F	NS	Hyperacusis
[30] Rahman 2017 Egypt	Case study	1, 5 yrs., M	ENT outpatients	Pain and intolerance of loud sounds
[46] Ralli 2018 Italy	Prospective cohort study	15, mean 5.8 yrs., MF	NS	Non-specific hypersensitivity to sound
[42] Ralli 2020 Italy	Comparative study	60, 4-12 yrs., MF	NS	Hyperacusis
[31] Rosing 2016 Denmark	Study 1: Prospective cohort study; study 2: Retrospective case review	Study 1:12,5-14 yrs., MF; study 2: 69,-yrs, MF	ENT or Educational Psychological Advisory Services (EPA) for children	Hyperacusis
[45] Sanchez 2019 Brazil	Case series	2, 12 yrs., 11 yrs., M	NS	Sound intolerance
[35] Shuper 2015 Israel	Case study	1, 9 yrs., M	Neurology clinic	Hypersensitivity to sound
[39] Wilson 2017 USA	Non-randomized trial	17 vs 13 control,no age for hyperacusis group, M	NS	Hyperacusis

The 21 included records were published between 1994 and 2020, and over half of them were case studies. Information on the type of care provider was included in ten records, four being Ear Nose and Throat (ENT) clinics [28–31], including one which specialised in paediatrics [24], and one Educational Psychological Advisory (EPA) service for children [31]. The rest included a Tinnitus and Hyperacusis Therapy Specialist Clinic [32], paediatric audiology [33], one tinnitus clinic [34], one neurology clinic [35], and two psychiatry clinics [36, 37], of which one [36] specialised in children and adolescents.

Specific complaints were reported in all 21 records. Although all records focused on hyperacusis, the term hyperacusis was used to describe the complaint in nine of those [21, 28, 33, 34, 38–42]. Other records used patient-specific complaints which feature in common definitions of hyperacusis. These include auditory hypersensitivity [36, 43], hypersensitivity to sound [35], ‘very sensitive’ and ‘oversensitive’ to sound [20, 37], ‘bothered and annoyed by sounds’ [44], ‘distress in response to ordinary sounds’ [29], ‘pain and intolerance of loud sounds’ [30], ‘pain and fear in response to sound’ in case 2 [2], ‘difficulty tolerating noise at school [2], and ‘sound intolerance’ [32, 45] (Table 1).

### Clinical profile

The clinical profile of children with hyperacusis was categorised in terms of the children’s age and gender, comorbid conditions, ULLs, troublesome noises, physical sensation, reaction, coping behaviours, and impact on functioning.

#### Age and gender

Three records included case studies of one male and two female children aged 5 years [30, 37, 38]. Three records reported on 11-year-old males and females [20, 36, 45], and two records reported on 9-year-old males [2, 35]. There were also cases of a 21-month-old female [29], a 6-year-old male [2, case 1], a 7-year-old female [41], a 12-year-old male [45], and a 15-year-old male [40]. The remaining records included both males and females, and reported age ranges of 4–7 years [46], 5–18 years [34], 5–12 years [44], 6–15 years [43], and 5–14 years [31, study 1]. One record only reported a median age of 7 years [28] and four records did not specify age of the hyperacusis group in their studies [31 study 2, 31, 32, 38]. Only one record [28] specified age at hyperacusis onset and that it was gradual in all 412 cases of the sample. Of the ten case studies included in this review, six were male [2, 30, 35, 36, 40, 45] and four were female children [29, 37, 41, 38].

#### Comorbid conditions

All but one record included children exhibiting comorbid conditions alongside hyperacusis. ASD was noted in seven records [20, 28, 33, 36, 37, 39, 40], tinnitus was reported in seven records [20, 31–34, 44, 45], five records reported William’s syndrome (WS) [29, 32, 33, 38, 43], four mentioned attention deficit hyperactivity disorder (ADHD) [10, 31, 33 study 1, 42] three records included patients with epilepsy [33, 35, 37] and three noted some degree of bilateral sensorineural or conductive hearing loss [28, 31, 33]. Two records included phonophobia [31, 44], auditory processing disorder [31, 33], and otitis media [33, 44]. Individual records also mentioned comorbid hypercalcemia [38], migraine [30], head trauma [31], cerebral palsy, Down’s syndrome, prematurity and post haemorrhagic hydrocephalus, dyspraxia, Klinefelter’s syndrome, Leigh’s syndrome, microcephaly and microdeletion, glue ear [33], and motion sickness [41].

#### Uncomfortable loudness levels (ULL/LDL)

Four records [32, 34, 44, 45] measured ULLs in children. Sanchez and Pereira [45] reported that these were 40–65 dB for case 1 and 80–90 dB for case 2 in both ears. Aazh et al. [32] found that the mean value of minimum ULL was 44.4 dB HL (SD = 6.8) in those categorised as having severe hyperacusis (ULL at any test frequency less than 30 dB HL), and 68.4 dB HL (SD = 12) in the rest of the group (diagnosed with hyperacusis but no ULL less than 30 dB HL for any test frequency). However the difference between the hyperacusis and the non-hyperacusis group was not statistically significant (*p* = .11). The rest of the records did not describe the values they obtained.

#### Troublesome noises

Thirteen records described noises that were reported as troublesome by children. Household electrical appliances [28, 36, 44] were the most commonly reported, especially vacuum cleaners [2, 31, 37, 39, 41], washing machines [33, 37], hand driers [33, 43], lawn mowers [2, 38], kitchen food processors [2], whistle/buzzer [44], toilet flushing [39], file alarms [43], drilling noises [33], radio or television [44, 45], telephone [29, 35, 38], and doorbells [33]. Other troublesome sounds included those typical to the school setting such as the school bell [2, 29, 42], music class [41], screams [42], teachers’ voices [45], classroom noise and school recess [42]. Traffic based sounds were also common such as sirens [33], and cars/traffic [33, 37, 45] as well as nature sounds including thunder [42], animals/insects [44], dog barking [33], and “fire crackers” [44]. More generally, loud music [38, 44] or loud sounds [30, 43, 44], especially if unexpected [43] or sudden [35] such as clapping [33], popping balloons and bombs [42] were reported as troublesome. Human-produced sounds we reported including crowds [38], the voices of children [36], and in particular laughing and high-pitched girls’ voices [40], babies [36, 43], family, friends, coughing and sneezing [40], restaurants [41] and snack bars [45].

#### Physical sensation

Physical sensation, i.e. the specific physical discomfort caused by the troublesome sound was reported as pain in five records [2, 30, 33, 38, 41], with one record [33] specifying this is experienced in the head or ears. Another record reported stomach pain and nausea that was concomitant with the painful noises [41].

#### Reaction

Children’s reactions to sounds were described in nine records. Covering their ears was the most common reaction [29, 33, 36–40, 43]. Others included crying [33, 37–40, 43], screaming [33], becoming verbally aggressive [40] or physically aggressive towards others [31, 33, 35, 40], or towards one self, e.g. hitting their own head [40]. Destroying items [38] was also mentioned. Two records [2, 37] described fear as a reaction. Others included symptoms such as sweaty palms, shaking, palpitations [43], headache, change in mood or facial expression [35], urinary incontinence, grinding one’s teeth, freezing, distress [33], running away [31, 37, 40], hiding [31], cringing, arching of the back [38], ‘going into meltdown’ [2], hyperactivity in noisy environments [46], or the child throwing themselves to the floor [40].

#### Coping behaviour

Five records described ways in which children reportedly cope with the troublesome noise. Two types of behaviour emerged. The more common was avoidance of places and activities [20] such as avoiding ‘the noisy corridors’ [40], ‘avoiding the noisy dinner halls’, or avoiding ‘public toilets in case someone switched the hand dryer on’ [33], ‘spending school recess in the silence of the library [45], or working ‘in the resource centre where it is quiet’ [40], ‘arriving in class once the other students have settled in their places’ [40], and sitting ‘in the very back of music class’ [41]. The second type of behaviour was wearing ear protection [20] such as noise reduction headphones [41]. Three records specified children wearing these all day long [45], at school or ‘when doing practical work in science and catering’ [40], or in the street and at home [2, 45].

#### Impact on functioning

The impact of hyperacusis on the child’s normal daily lives was defined in eight records. Based on the information provided in these records, the children were limited in three main areas – getting out of the house, school life, and in social and recreational settings.

Difficulties in getting out of the house were described in four records [33, 36, 37, 43]. Adanir et al. [36] specified that the child ‘refused to go out except to school’. Another record stated that the child did not tolerate walking in the street [37], and in another the child did not want to leave the house [43]. One record noted that when the child did leave the house, they had toileting accidents due to avoiding public toilets because of electric hand dryers [33]. Another child’s family had to keep stopping the car on the road every time a vehicle passed [33].

In terms of school life, two records noted avoiding school when testing fire alarms [43], or sometimes avoiding school altogether [2]. Two records reported that when the children were in school, they felt disturbed [36] or anxious [2]. Four records alluded to children generally having difficulties or severe limitations at school [33, 35, 40, 45]. One record specified that the child ‘could not hold a pencil to write because he was covering his ears’ [36], and another reported ‘limitations especially during physical education when sports activities were practiced indoors’ [45]. Aazh et al. [40] described a child with hyperacusis who was disturbing other students’ learning ‘by shouting at them to be quiet’ and disturbing their concentration during exams. The child was also unable to attend noisy lessons such as drama and physical education.

Three records noted child and family social and recreational struggles [33, 35, 37]. Two records provided specific examples, i.e. the ‘mother has problems using the vacuum cleaner’ [37], ‘avoiding parties’ [33], and having to whisper ‘happy birthday’ at the child’s own party [33].

### Assessment

Methods of assessing hyperacusis were reported in 14 records (Table 2). Methods included neurological assessment, the use of ULLs, semi-structured interviews, questionnaires and observation.

**Table 2:** Methods of assessing hyperacusis in children

Study Reference	Assessment Method	Description
[20]	Semi-structured interview	The child was asked whether he/she ‘ever experiences over-sensitivity or distress to a particular sound’. If ‘yes’ then a further question was ‘whether they stay away from places or activities because of sensitivity to sounds’
[31]	Questionnaire	A structured email questionnaire - not specified
[32]	ULLs	ULLs using the recommended BSA procedure with modifications
[33]	Semi-structured interview	Children were asked ‘if they were troubled/ bothered by these symptoms, which noises were particularly troublesome and how they react to them’
[34]	ULLs	Frequency 1.2 and 4 kHz
[37]	Visual Analogue Scale (VAS)	Parent completed 10 cm long line with anchors ‘no hyperacusia’ and ‘worst possible hyperacussia’
[38]	Questionnaire	The William’s Syndrome Questionnaire
[39]	Semi-structured interview	Questions from published hyperacusis questionnaires eliciting recall of various attributes of hyperacusis and defensiveness such as troublesome sounds and associated behavioural responses
[41]	Neurological assessment	512 tuning fork, 128 tuning fork
[42]	Semi-structured interview and parental questionnaire	Semi-structured interview: children were asked if they were bothered by sounds and if so, to choose which ones were bothersome from a list of options. A multiple choice parental questionnaire examining children’s hypersensitivity to sounds and their reactions.
[46]	Observation and questionnaire	Observation: sounds were presented on speakers and the children’s reactions recorded Questionnaire: four multiple choice items adapted from Coelho 2007.
[43]	Semi-structured interview and a questionnaire	Interview: questions not specified Questionnaire: Sensory Profile (short form)
[44]	Semi-structured interview and ULLs	Interview: ‘If a positive answer was given to the question ‘Are you bothered by any kind of sound or noise?’ and the description of this sound and were able to identify at least 10 sounds from a list of 20 sounds, the responses were classified as ‘being annoyed by specific sounds’. ULLs – maximum audiometer output 110 dB HL at 0.25 Hz, 120 dB HL from 0.5HZ to 6.0 kHz and 100 dB at 8.0 kHz.
[45]	ULLs with history of intolerance to certain sounds	ULLs measured at least to 500, 1000, 2000 4000 Hz

#### Neurological assessment

Neurological assessment was conducted in one record [41]. The assessment was conducted using 512 and 128 Hz tuning forks. Tuning fork tests were used to assess sound or vibration-induced pain at or near the ear.

#### ULLs

Another method of assessment was by taking ULLs [32, 34, 44, 45] and only one of the four records who used it [32] specified that they used the BSA approved method [47].

#### Semi-structured interviews

A more common method of assessing hyperacusis was through semi-structured interviews. This method was used in six of the records [20, 33, 39, 42–44] with questions to be answered by the children and/or their parents. The types of questions asked in different records varied. One record specified the question asked as ‘are you bothered by any kind of sound or noise?’ followed by checking their ability to select at least 10 bothersome sounds from a pre-specified list of 20 sounds [44]. In Hall et al. [20] children were asked if they ‘ever experience oversensitivity or distress to particular sounds’ and if so, ‘whether they stay away from places or activities because of sensitivity to sound’. In Myne et al. [33] children were asked ‘if they were troubled or bothered by these symptoms, which noises were particularly troublesome, and how they react to them’. Ralli et al. [42] included four questions, one of which focused on hyperacusis, namely “Are you bothered by any kind of sound or noise?” with a further question to clarify the troublesome sounds from a list of options including: "School recess, TV, Car, Toys, Firecrackers, Classroom noise, Radio, Motorcycle, Balloons, Bombs, Screams, Mixer, Truck, Whistle, Thunder, School bell, Telephone, Ambulance, Musical instruments, Dogs. Wilson et al. [39] used questions from published auditory and hyperacusis questionnaires [48, 49] to inform the questions asked in the semi-structured interviews. The questions encouraged children to describe the troublesome sounds and their reactions to these. They also used an adapted version of Knudson’s et al. [50] evaluation process, whereby a score of five or more was interpreted as severe hyperacusis. The authors provided no further detail on the questions used. One record did not specify the interview questions asked [43].

#### Questionnaires

Six records used questionnaires to assess paediatric hyperacusis though two of them were designed for WS rather than hyperacusis. Janes et al. [43] used the Sensory Profile Short Form, designed for patients with WS. One item asks parents to assess the child’s auditory hypersensitivity on a 5-point Likert scale from 'never' to 'always'. Children with scores of 'sometimes' and 'always' were classed as having auditory hypersensitivity. O’Reilly et al. [38] used the William’s Syndrome Questionnaire by Klein et al. [51] to assess hyperacusis, providing no further information on the questionnaire items used. Ghandizeh et al. [37] specified use of a hyperacusis-specific parent-completed Visual Analogue Scale (VAS), a 10 cm line with anchors 'no hyperacusia' and 'worst possible hyperacusia'. Rosing et al. [31] used a questionnaire but did not specify which. Ralli et al. [46] used a questionnaire which he adapted from the questions previously used in Coelho et al. [44, 52]. The adapted version used in Ralli et al. [46] had four multiple choice items. A score of eight points or more was considered positive for hyperacusis. Ralli et al. [46] also used observation in conjunction with the questionnaire to assess hyperacusis. As part of this, specific sounds were presented to the children at regular intervals. Children were considered to have hyperacusis if they displayed at least one of the reactions listed on a form including "cover ears with hands; cries; escapes from the sound source; tries to avoid sound; says "hurts ears"; says "I don’t like it'. Ralli et al. 2020 [42] used a two-part parental questionnaire. Part one consisted of four short and concise multiple-choice questions investigating their children’s relationships with sounds.

Part two included six questions investigating the children’s most common reactions to these sounds. A diagnosis of hyperacusis was given using the combined scores from the child interview (described in the semi-structured interviews section) and the parental questionnaire. Both the parental questionnaire and the child interview were modifications from the work by Coelho et al. 2007 [44].

### Treatment and outcomes

Nine records reported on a treatment for hyperacusis (Table 3). Four of those describe some form of psychological therapy alone or in combination with sound therapy or medication [2 case 2, 28, 31, 46 case1]. Three used medication alone as treatment [30, 35, 37]. Sound therapy was evaluated alone [2 case1], and in conjunction with medication (46 case 2), one reported on the use of TRT [34], and one utilised neuro-rehabilitation [41].

**Table 3:** Treatments and outcomes

Study Reference	Treatment	Outcome
[2]	Case 1: WNGs Case 2: CBT	Case 1: ‘ability to cope was much improved’; case 2: ‘improvement seen two weeks later in resisting troublesome sounds’
[28]	Behavioural therapy and Pure Relaxation Therapy Ball for home use	25% had sufficient improvement to permit discharge; 3% required more than three sessions before symptom resolution; 1% were referred back to service; 25% did not attend treatment
[30]	Topamax 25 mg tablets	Patient reported better tolerance to loud sounds
[31]	Counselling	Information and coping strategies
[34]	TRT	Rapid remission of hyperacusis; 75% showed significant improvement after 2–3 months, the rest – after 6 months.
[35]	Valproic acid 15 mg/kg alone then combined with risperdone 0.5 mg/day	Some improvement; could attend school but remained incapacitataed by sudden and loud sounds
[37]	Sodium valproate 600 mg/day and risperdone 0.5 mg/day	Improvement on VAS from 10 down to 4 or 5.
[41]	Neuro-rehab	Vestibular and utri-circular activation; gaze stabilization and macro-saccades; passive complex motion and home exercises to promote neuro-plascticity
[45]	Case 1: Counselling and Gingko biloba Case 2: Gingko biloba and environmental sounds	Case 1: patient reported gradual and stable improvement Case 2: patient reported stable improvement

#### Psychological therapy

Kennedy et al. [2, case 2] described a 9-year-old male treated with Cognitive Behavioural Therapy (CBT), including identifying a hierarchy of sounds and desensitization through graded step-by-step exposure to sounds, with rewards chosen by the child. Rewards in this case were picture cards of favourite football players. Desensitisation was practiced daily with the child’s mother. Other treatment components included relaxation and use of positive thoughts such as ‘I can do this’ and ‘I’m brave’. Improvement was noted 2 weeks later with the child being able to tolerate the sound of hoovers and the school bell.

Amir et al. [28] described behavioural therapy combined with using a pure-tone relaxation therapy ball for home use. The authors provided no further details about the treatment. Twenty-five percent of children were ‘considered to have sufficient symptom improvement’.

Rosing et al. [31] reviewed the practices of treatment centres where children in the range of 5 to 14 years old received counselling, in the form of information and coping strategies, and sound therapy. The authors described no further details about the treatment content and duration, and no outcomes of the treatment were provided. In Sanchez et al. [46 case 1] a 12-year-old male was treated with counselling, including information about the condition, its aetiology and association with tinnitus. This was combined with Gingko biloba extract (80 mg twice a day for 2 months initially). No further information was provided about the treatment. The authors noted gradual improvement in hyperacusis which was stable in the long term.

#### Medication

In Ghandizeh et al. [37] improvement in hyperacusis in terms of VAS scores from 10 (max) to 4/5 was seen using Risperdone (0.5 mg per day) however the effect did not appear to be stable. Rahman et al. [30] reported on a treatment for migraine using Topamax 25 mg tablets (no further dosage provided) with the child reporting ‘better tolerance to loud sounds’. Shuper et al. [35] reported treatment with valproic acid in a dose of 15 mg/kg and Risperdone (0.5 mg/d). The authors reported a ‘marked improvement’ and being able to attend school though he was still severely ‘incapacitated by sudden and loud noises’.

#### Tinnitus retraining therapy (TRT)

Borawska et al. [34] reported a case note review of tinnitus and hyperacusis children treated with ‘hyperacusis TRT’. Although TRT [53] was originally designed for tinnitus, it can be suited to treat hyperacusis as the two conditions are often co-occurring. The components of this treatment were not described in the record but likely included directive counselling and sound therapy in the form of enriched sound environment achieved via sound generators or combination (sound generator and hearing aid) instruments [54]. This is important in order to achieve habituation whereby the patient no longer notices and reacts to the bothersome sounds. Seventy-five percent of children with hyperacusis showed significant improvement after 2–3 months, the other 25% showed improvement after 6 months.

#### Sound therapy

Sound therapy, involving white noise generators (WNGs), was used with one case in Kennedy et al. [2, case1]. Devices were set at an output of 55 dB SPL to be worn all day at school. The child found ‘his ability to cope much improved’, e.g. ‘when there was building work near his classroom he was still able to comfortably take part in lessons’. Sanchez et al. [46, case 2] also described a child treated with environmental sounds, combined with taking Gingko biloba extract. After 3 months there was gradual improvement in behaviour when facing sounds, with the child ‘rarely using ear protection’.

#### Neuro-rehabilitation

Esposito and Elkins [41] described a neuro-rehabilitation for hyperacusis which was designed to also treat the co-occurring nausea experienced by one child. It comprised of vestibular activation with leftward rotations in a chair; divergence eye exercises with utricircular stimulation in a posterior direction; gaze stabilization exercises and microsaccades to small targets. Exercises were also performed at home to enhance neuroplasticity. Improvement was observed from the day after treatment with the child being able to attend music class without using ear protection. The child remained pain and nausea free from the tuning fork and other noises five visits later.

## Discussion

This scoping review aimed to catalogue the literature providing a clinical profile of children experiencing hyperacusis, and how they have been assessed and treated. Only 21 relevant records were identified, indicating a paucity of research in this field. We mapped the available evidence on the children’s clinical profile of hyperacusis in terms of age and gender, comorbid conditions, LDLs, troublesome sounds, physical sensations, reactions, coping behaviours, and areas of impairment in daily living. Relevant assessment and treatment methods used with children with hyperacusis were also catalogued.

Age at time of hyperacusis onset was not reported in the records we reviewed, however one study which involved a large sample indicated that all children had a gradual onset [28]. In terms of age at presentation, we found that a few of the case studies reported on 5- and 11- year-olds. However, studies with larger samples reported that the commonest age at presentation as 3 to 4 years old [33, 55]. In clinical practice, hyperacusis in children between 3 and 4 years old is considered to occur as part of normal auditory development, which is likely to settle by itself with the maturation of the central auditory system [56, 57].

In terms of gender, the majority of the case studies we reviewed described male children. Although this does not signify that hyperacusis is more prevalent in males - studies with larger samples have reported a male majority [28, 33, 55]. This could be due to the type of research population selected in those studies. ASD for example, where hyperacusis is a common complaint, is more common in males. On the other hand, Ralli et al. [46] observed a majority of male children in their sample and this difference was found to be statistically significant. More research is needed to clarify the potential gender effect.

As already noted, hyperacusis is often a symptom in ASD [18] and indeed, we found that ASD was one of the two most commonly reported comorbid conditions of hyperacusis. Tinnitus was just as common in children with hyperacusis, followed by WS and ADHD. Epilepsy and hearing loss were also commonly reported. Across the records we reviewed the proportion of children who experienced primary hyperacusis, in isolation of other health conditions, was smaller. Of these children, some have experienced glue ear, which can increase the perception of loudness once it has resolved [33]. This highlights the question whether hyperacusis is a symptom or a disease in itself, to be addressed by future aetiology research, not only of hyperacusis, but also ASD, WS and tinnitus, and their association.

Although natural variety exists [3], patients with hyperacusis are generally presumed to have lowered ULLs [20] and taking ULLs had become the traditional tool for diagnosing hyperacusis. Surprisingly however, the majority of records did not assess ULLs. The few that did, reported them in little detail. These findings reflect the recent suggestions that ULLs are not a reliable measure in adults or children [58, 59]. Further to this, measuring ULLs can also be distressing for young children and can undermine the building of rapport with the clinician [60]. This could be a consideration for standard ENT assessment for hyperacusis.

There was a variety of sounds reported by children as troublesome. From the data catalogued here it appears that hyperacusis is generally associated with ‘unnatural’ noise produced by machines, or with natural sounds, e.g. those produced by people or animals, that are simultaneously loud and sudden. It is worth noting that children are not bothered by such sounds if they are produced by themselves which implies that it is not just certain bothersome sounds that create a reaction but perhaps also the lack of control the child feels over them. Indeed Phillips and Carr 1998 [61] proposed that issues with sound perception may arise from both physical and psychological sources. Psychological or emotional factors in the hyperacusis reaction and their interplay with the physiological responses must be addressed in future research into the aetiology of hyperacusis.

It is also possible that patients with developmental disorders are more likely to be troubled by a particular type of sound than others with hyperacusis. Future research on the clinical profile of paediatric hyperacusis could provide clarification on the ways in which children with and without developmental disorders differ, and the characteristics of sounds that bother them. Such information would be useful to better define the symptomatology of paediatric hyperacusis as an independent condition rather than as being part of a condition or syndrome.

There is little literature on the physical sensation of hyperacusis, i.e. what makes the sound so uncomfortable. We found that where there were reports of physical sensation, those were of ‘pain’ in the ears, and in one case in the head. In another case, co-occurring nausea was also observed. The reasons for such physical sensations could be a consideration for research into the aetiology of hyperacusis.

Although the literature describes a wide variety of behavioural reactions, we found that most commonly children covered their ears, cried, or became aggressive. Aggression in children is a common expression of anxiety [62]. Older children were more likely to cope by avoiding the situations where they might be exposed to the sound, or wearing ear protection. Younger children were less likely to engage in coping behaviour; instead, their parents had put limitations on their usual activities to avoid their child becoming distressed. The biggest impact on family functioning was in terms of getting out of the house, attending social events, and school performance. The first two would limit social interactions with peers, and the normal life of the child’s family members causing tension in their relationships. All three factors can result in high levels of stress, low quality of life, and hinder progression in life for both child and family.

Unfortunately as this condition has emerged recently and has a poor evidence base concerning children, clinicians such as GPs, paediatricians, school nurses, psychologists, audiologists, educators, and health visitors, who encounter this condition, are unprepared to support such families. This originates in the current lack of child-specific assessment and treatment tools. None of the records used validated hyperacusis - specific assessment questionnaires that were standardised with a paediatric population. Semi-structured interviews were commonly used to diagnose and assess the severity of hyperacusis. However, there was notable variation in the types of questions the children or their parents were asked. A validated questionnaire standardised with a paediatric population is urgently required to assist in the early identification of children with significant hyperacusis and to support clinical research evaluating the effectiveness of interventions.

None of the records we identified sought to develop a treatment specifically tailored for children with hyperacusis and those that described treatments used for children had no previous empirical evidence of effectiveness. There was also a great variability in the treatments documented and in the different combinations used e.g. psychological treatment with medication or with sound therapy. However, none of the records provided a detailed description of its treatment components and none demonstrated significance in their patient outcomes. Randomised controlled trials are required to compare and assess the effectiveness of these treatments for children. Appropriate treatment and assessment could help identify and support such children both in school and at home. There has been positive development in the field of tinnitus where there is designated clinical guidance for managing children and potential adjustments in the classroom [22]. Future research could focus on adapting these for children with hyperacusis.

Some limitations of this review are noted. Although we used a wide range of synonyms of hyperacusis in our searches, there may have been relevant studies that have not been identified. In terms of the origin of the studies we reviewed, there was a dominance of records from western countries resulting in a potential bias of our findings. For instance, the clinical profile in other cultures may have different dominant features, which future research could explore. In addition to this, the majority of children that were described had some type of a developmental condition, which may have further biased the clinical profile we describe. The records obtained may also not represent the full clinical profile of hyperacusis due to the potential presence of the ‘file drawer effect’, where certain case studies, for example those with children without co-occurring conditions, were not published. A further limitation to this review is that it is limited to research published in the English language only. As a result, it is possible that our findings may present with cultural and ethnic bias. Specifically the clinical profile, assessment and treatments methods may differ in other countries. The relationship between hyperacusis in children and cultural factors would be interesting to assess in future research. Another limitation to this review is that there is no standardised diagnostic criteria for hyperacusis. For consistency we only included records that explicitly used the term hyperacusis, but the underpinning diagnostic criteria may differ across cases hence there may be instances of misdiagnosis.

This scoping review catalogued the existing research on the clinical profile of hyperacusis in children and the methods of assessment and treatment. The clinically relevant findings emerging from this review, which would need further verification in research, are the following: the commonest presentation age is 3 to 4 years old with a potential male dominance; ASD and tinnitus were the most common co-occurring conditions; the most common troublesome noises were household electrical appliances; the common reaction was to cover their ears, cry and aggressive behaviour. The commonest impact of hyperacusis was on family functioning, including getting out of the house and attending social events and on school performance. Semi-structured interviews and questionnaires were the most common assessment method. All four treatment options for hyperacusis in children – psychological, sound, medication, TRT and neuro-rehabilitation, were applied either alone or in combination and all showed notional improvement in severity.

## Conclusion

This review aimed to bridge a gap in our understanding of the ways in which hyperacusis presents in children. The information we catalogued on various elements of clinical profile can serve as a stepping stone in specifying the symptoms of paediatric hyperacusis, and aid clinical diagnosis. It will also support future research to develop questionnaires for clinical measurement of the impact of hyperacusis on children, and the measurement of treatment related change in clinic and in trials.

## Data Availability

The datasets used and/or analysed during the current study are available from the corresponding author on reasonable request.

## Abbreviations

ADHD: Attention Deficit Hyperactivity Disorder
AS: Asperger’s Syndrome
ASD: Autism Spectrum Disorder/s
BSA: British Society of Audiology
ENT: Ear Nose and Throat
EPA: Educational Psychological Advisory
TRT: Tinnitus Retraining Therapy
ULLs: Uncomfortable Loudness Levels
WNGs: White Noise Generators
WS: William’s Syndrome
